# Exploring expectation effects in EMDR: does prior treatment knowledge affect the degrading effects of eye movements on memories?

**DOI:** 10.1080/20008198.2017.1328954

**Published:** 2017-06-19

**Authors:** Marianne Littel, Kevin van Schie, Marcel A. van den Hout

**Affiliations:** ^a^ Institute of Psychology, Erasmus University Rotterdam, Rotterdam, the Netherlands; ^b^ Department of Clinical Psychology, Utrecht University, Utrecht, the Netherlands

**Keywords:** EMDR, eye movements, expectancy effects, prior knowledge, Bayesian approach

## Abstract

**Background**: Eye movement desensitization and reprocessing (EMDR) is an effective psychological treatment for posttraumatic stress disorder. Recalling a memory while simultaneously making eye movements (EM) decreases a memory’s vividness and/or emotionality. It has been argued that non-specific factors, such as treatment expectancy and experimental demand, may contribute to the EMDR’s effectiveness.

**Objective**: The present study was designed to test whether expectations about the working mechanism of EMDR would alter the memory attenuating effects of EM. Two experiments were conducted. In Experiment 1, we examined the effects of pre-existing (non-manipulated) knowledge of EMDR in participants with and without prior knowledge. In Experiment 2, we experimentally manipulated prior knowledge by providing participants without prior knowledge with correct or incorrect information about EMDR’s working mechanism.

**Method**: Participants in both experiments recalled two aversive, autobiographical memories during brief sets of EM (Recall+EM) or keeping eyes stationary (Recall Only). Before and after the intervention, participants scored their memories on vividness and emotionality. A Bayesian approach was used to compare two competing hypotheses on the effects of (existing/given) prior knowledge: (1) Prior (correct) knowledge increases the effects of Recall+EM vs. Recall Only, vs. (2) prior knowledge does not affect the effects of Recall+EM.

**Results**: Recall+EM caused greater reductions in memory vividness and emotionality than Recall Only in all groups, including the incorrect information group. In Experiment 1, both hypotheses were supported by the data: prior knowledge boosted the effects of EM, but only modestly. In Experiment 2, the second hypothesis was clearly supported over the first: providing knowledge of the underlying mechanism of EMDR did not alter the effects of EM.

**Conclusions**: Recall+EM appears to be quite robust against the effects of prior expectations. As Recall+EM is the core component of EMDR, expectancy effects probably contribute little to the effectiveness of EMDR treatment.

Eye movement desensitization and reprocessing (EMDR) has been extensively validated as an effective psychological treatment for posttraumatic stress disorder (PTSD; Bisson et al., 2007; Bradley, Greene, Russ, Dutra, & Westen, 2005; Chen et al., 2014). It involves focusing simultaneously on traumatic memories along with associated thoughts, emotions, and bodily sensations, as well as bilateral stimulation, generally in the form of horizontal eye movements (EM). A recent meta-analysis shows that these EM are crucial, as they have a significant, additive memory degrading effect to mere exposure to the traumatic memory (Lee & Cuijpers, 2013).

Currently, the most evidenced account for the beneficial effects of EM is provided by the working memory (WM) theory (Andrade, Kavanagh, & Baddeley, 1997; Maxfield, Melnyk, & Hayman, 2008; van den Hout & Engelhard, 2012). WM is a cognitive system for temporal storage and manipulation of information (Baddeley, 2012). It has been consistently demonstrated that performance deteriorates when two tasks make demands on the same WM resources, indicating that WM has limited capacity. Focussing on a memory (van Veen et al., 2015) and engaging in EM, both tax WM resources (Engelhard, van Uijen, & van den Hout, 2010; van den Hout et al., 2011, 2010). Simultaneously performing these tasks therefore reduces the sensory quality of the memory, making it less vivid and less emotional. It is likely that, after EMDR, the less rich, degraded memory is reconsolidated into long-term storage which may explain long-term effects (Gunter & Bodner, 2008; Nader & Hardt, 2009; van den Hout & Engelhard, 2012).

The memory degrading effects of EM, the core intervention of EMDR, are reproducible in a laboratory setting (van den Hout & Engelhard, 2012; van den Hout, Muris, Salemink, & Kindt, 2001). During a procedurally simple ‘EMDR-lab model’, healthy participants recall aversive autobiographical memories and rate them in terms of vividness and emotionality. Then, the memories are recalled while tracking a moving dot on a computer screen, which induces horizontal eye movements (Recall+EM), or memories are recalled while keeping the eyes still (control condition; Recall Only). Afterwards, both memories are again recalled and rated on vividness and emotionality. Using this laboratory design, different variables can be manipulated in order to examine the underlying working mechanisms of EMDR. Studies that have adopted the model have shown, for example, that memories are not only degraded by EM, but also by other WM taxing dual tasks, such as complex counting (van den Hout et al., 2010) and mindful breathing (van den Hout et al., 2011), and that not only negative memories can be altered by EM, but also positive memories (Engelhard et al., 2010; Hornsveld et al., 2011; Littel, van den Hout, & Engelhard, 2016), providing evidence for the abovementioned WM account.

As with all biomedical and psychological interventions, the question arises to what extent the high effectiveness of EMDR can be attributed to non-specific factors, such as treatment credibility and expectancy. Both psychotherapy outcome expectancy and treatment credibility are shown to be positively related to treatment outcomes (Constantino, Arnkoff, Glass, Ametrano, & Smith, 2011; Taylor, 2003). With regard to EMDR specifically, previous authors have asserted the view that beneficial effects of the treatment are incidental and might be explained by credibility, expectation for improvement, experimental demand, therapist enthusiasm, and therapist allegiance (Devilly, 2005; Herbert et al., 2000; Lohr et al., 1992; Lohr, Lilienfeld, Tolin, & Herbert, 1999).

Within the EMDR-lab model, non-specific intervention effects are by default controlled for by excluding participants with detailed prior knowledge of EMDR’s effectiveness and/or the underlying mechanism. Hence, the commonly observed superiority of Recall+EM over Recall Only cannot be attributed to positive expectancies of the EM manipulation. Nevertheless, whether prior knowledge of EMDR truly affects the results remains an empirical question. Addressing this question is relevant for EMDR research, as it will indicate whether it is necessary to exclude participants with prior knowledge. Most importantly, however, it will reveal whether expectancy effects might contribute to the beneficial effects of EMDR treatment, which has high clinical relevance.

Therefore, in Experiment 1 we tested two pre-specified and *competing* hypotheses regarding the role of non-experimentally manipulated, prior knowledge of EMDR on the effects of Recall+EM vs. Recall Only: (1) prior knowledge strengthens the decreases in memory vividness and emotionality after Recall+EM vs. Recall Only; and (2) prior knowledge does not affect memory degrading by Recall+EM vs. Recall Only. We used an experimental design, and included individuals with and without prior knowledge of EMDR. We used a Bayesian approach to critically test which of the two hypotheses is most likely.

## 1. Ethics statement

The research was conducted according to the principles expressed in the Declaration of Helsinki. In both experiments in this article, healthy human participants were tested. All participants provided written informed consent. In giving their consent, participants acknowledged to have read and to have agreed with the rules regarding participation, and the researchers’ commitments and privacy policy. Participants were informed that they could stop the experiment at any time without the need to provide a reason for stopping. All gathered data were analysed anonymously. Afterwards participants were debriefed.

## 2. Experiment 1

### 2.1. Methods

#### 2.1.1. Participants

Forty-three individuals (*M*_age_ = 21.58, *SD*_age_ = 1.87, 9 males, 34 females) participated in the first study. Inclusion was limited to individuals over 18 without current self-reported psychopathology, and who reported never to have received EMDR therapy. Participants were mainly students recruited at Utrecht University. Based on their self-reported specific knowledge of the memory degrading effects of EMDR and/or its proposed underlying working mechanism, they were divided into a ‘prior knowledge group’ (*n* = 22) or a ‘no knowledge group’ (*n* = 21). See Table 1 for the specific inclusion criteria. See Table 2 for demographics per group. All participants provided written informed consent and received course credit or financial reimbursement.

**Table 1.** Criteria for inclusion in the prior knowledge and no knowledge groups.

Prior knowledge	No knowledge
*Participant describes that…*	Participant has never heard of EMDR; or
* because of EMDR memories become less vivid/clear/intense/emotional/negative/unpleasant; or	Participant has heard of EMDR but cannot describe what it is or gives incorrect description; or
* after EMDR memories are more distant/vague/blurry; or	*Participant knows that…*
* after EMDR memories are erased/less accessible/difficult to recollect; or	* EMDR is ‘a psychological therapy’ or ‘a therapy to treat trauma/PTSD’; or
* because of EMDR memories change/are altered/updated/overwritten; or	* EMDR has something to do with eye movements/beeps/clicks, but nothing more; or
Participant describes (parts of) the working memory theory.	* EMDR is a psychological therapy (for PTSD) during which eye movements are made (or beeps/clicks are presented), but nothing more.

**Table 2.** Demographics per knowledge group.

	Prior knowledge	No knowledge
*n*	22	21
Mean age (*SD*)	21.73 (1.88)	21.43 (1.89)
Gender (male, female)	1, 21	8, 13
Ethnicity (Dutch, other)	21, 1	20, 1
Education (higher, lower)	22, 0	20, 1
Psychology student (yes, no)	18, 4	8, 13

#### 2.1.2. Materials and procedure

At the start of the experiment, participants were instructed to select two aversive, autobiographical memories and grade the emotional intensity of the memories on a scale from 1 to 10. If memories were graded < 6 or > 9, they were considered either not aversive enough or too aversive, and participants were instructed to select a different memory. In line with the Dutch EMDR protocol (de Jongh & Ten Broeke, 2012), they had to ‘play’ these memories in their minds as vividly as possible and take a ‘screenshot’ of the most emotionally intense moment. Participants labelled the resulting images with a keyword, which was used to refer to the selected memories in the remainder of the experiment. For counterbalancing purposes, participants then ranked the images based on emotional intensity.

A computerized dual taxation task was used to simulate the EM component of EMDR. Participants were instructed to recall one of their aversive memories. Meanwhile they had to track a horizontally moving dot (1 Hz) on a black screen (Recall+EM), or watch a black screen without a dot (Recall Only). The moving dots and blank screens were displayed during six intervals of 24 s separated by 10 s breaks (cf. van Schie, van Veen, Engelhard, Klugkist, & van den Hout, 2016; van Veen et al., 2015). Before (pretest) and after (posttest) each intervention participants recalled the aversive memory for 10 s and rated it on vividness and emotionality using Visual Analog Scales (VASs) ranging from 0 (not vivid/not unpleasant) to 100 (very vivid/very unpleasant). The experimental task was programmed in and presented with OpenSesame (Mathôt, Schreij, & Theeuwes, 2012). Participants were seated approximately 50 cm from the screen.

#### 2.1.3. Data analysis

Data were analysed with the BIEMS software package, which uses a Bayesian model selection criterion (see Mulder et al., 2009; Mulder, Hoijtink, & de Leeuw, 2012; Mulder, Hoijtink, & Klugkist, 2010). BIEMS evaluates the relative likelihood of the data for different competing hypotheses, which is expressed as a Bayes Factor (BF; Kass & Raftery, 1995). BIEMS specifically computes a BF for a constrained hypothesis against an unconstrained hypothesis. A BF of 1 means that there is equal support for a specified constrained hypothesis and the unconstrained model; one does not outperform the other. BF > 1 indicates that the study hypothesis outperforms the unconstrained model, whereas BF < 1 means the opposite. It is possible to directly compare BFs of different models, when each constrained BF is calculated against the same unconstrained model. Because we were interested in evaluating the relative likelihood of the data under different competing hypotheses, we did not use null hypothesis significance testing (NHST). In NHST one can only gather evidence against the null hypothesis, but never in favour of the null hypothesis, which makes evaluating hypotheses within the current study and within the field of experimental psychopathology largely unsuitable (Krypotos, Blanken, Arnaudova, Matzke, & Beckers, 2016).

In the current study, there were two groups (prior knowledge and no knowledge) and two intervention conditions (Recall+EM and Recall Only). Assessment of memory vividness and emotionality took place before the interventions (pretest) and immediately after the interventions (posttest). Pre-post difference scores were calculated for vividness and emotionality ratings in Recall+EM and Recall Only conditions, with higher scores indicating larger decreases in response to an intervention. The following two *competing* hypotheses were compared: (1) prior knowledge strengthens decreases in memory vividness and emotionality after EM compared to Recall Only; and (2) prior knowledge does not affect effects of EM relative to Recall Only on memory vividness and emotionality. See Table 3 for the constraints of both hypotheses.

**Table 3.** Hypothesis constraints for vividness and emotionality (pre minus post) difference scores for the hypotheses for the group with prior knowledge and the group with no knowledge. EM = Eye Movements.

Hypothesis 1	Both groups: EM > Recall Only
	(EM _prior knowledge_ – Recall Only _prior knowledge_) > (EM _no knowledge_ – Recall Only _no knowledge_
Hypothesis 2	Both groups: EM > Recall Only
	(EM _prior knowledge_ – Recall Only _prior knowledge_) = (EM _no knowledge_ – Recall Only _no knowledge_)

### 2.2. Results

**Figure 1.**
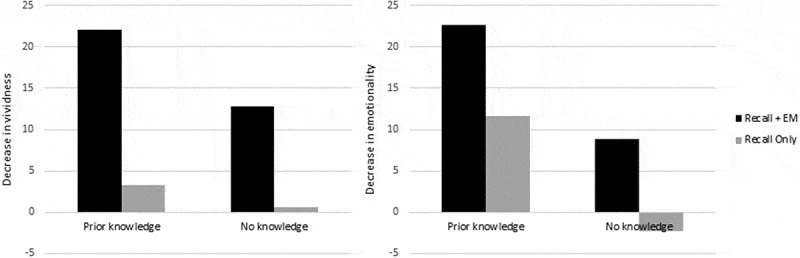
Mean decreases in memory vividness (left) and emotionality (right) after Recall+EM and Recall Only for the prior knowledge and the no knowledge group.

Figure 1 suggests that the observed data patterns for memory vividness are in line with hypothesis 1. Bayesian analyses showed a BF of 4.99 for hypothesis 1, and a BF of 3.40 for hypothesis 2. Therefore, both models are supported by the data, but model 1 appears to be somewhat (1.5 times) more likely than model 2. This is confirmed by the raw data-difference scores (i.e., the decrease after Recall+EM minus decrease after Recall Only), showing a slightly larger difference for the prior knowledge group (*M_dif_* = 18.74) than for the no knowledge group (*M_dif_* = 12.18).

For memory emotionality, Bayesian analyses showed a BF of 3.91 for hypothesis 1, and a BF of 4.85 for hypothesis 2. Again, both models are supported by the data, although model 2 appears to be slightly (1.2 times) more likely than model 1 (see Figure 1). This is confirmed by the raw data-difference scores, showing no approximately equal differences for the prior knowledge group (*M_dif_* = 11.01) and the no knowledge group (*M_dif_* = 11.07). See Appendix for mean (*SD*) vividness and emotionality decreases per group.

### 2.3. Discussion

The aim of this first experiment was to test whether pre-existing knowledge of the proposed mechanism of EMDR would increase the commonly observed, memory degrading effects of EM in a laboratory setting. The results confirmed that Recall+EM was superior to Recall Only in decreasing memory vividness and emotionality, which is in line with the findings of many previous studies (e.g., Leer, Engelhard, & van den Hout, 2014; van Schie et al., 2016; van Veen et al., 2015; van Veen, Engelhard, & van den Hout, 2016; and see van den Hout & Engelhard, 2012). Furthermore, it was demonstrated that prior knowledge increased the effects of Recall+EM on memory vividness, but did not affect the effects of Recall+EM on memory emotionality. This means that both our hypotheses are partly confirmed. Note, however, that neither for memory vividness nor for memory emotionality, the hypotheses unambiguously outperformed each other.

The contradictory observation that both hypotheses are supported by the data can be explained by the reductions in memory emotionality after Recall Only (see Figure 1, grey bars). Because previous studies only show small reductions (e.g., Gunter & Bodner, 2008) or even increases in emotionality after Recall Only (e.g., Engelhard et al., 2010; van den Hout et al., 2001), these reductions are unexpectedly high. It is evident from the data that the *actual* decrease in emotionality for Recall+EM is larger for the prior knowledge group (*M* = 22.60) compared to the no knowledge group (*M* = 8.80; see Figure 1, black bars). However, because both models encompassed constraints that defined decreases in Recall+EM in relation to decreases in Recall Only, the *relative* decrease was not larger for the prior knowledge group (*M_dif_* = 11.01) compared to the no knowledge group (*M_dif_* = 11.07; see Figure 1, black bars vs. grey bars).

Although the role of prior knowledge seems to be relatively small, the results suggest that, in future experimental studies investigating EMDR components, individuals with prior knowledge of the underlying mechanism of EMDR should (continue to) be excluded from participation, at least when a between-group design is adopted.

It is important to note that the current study was not randomized. Therefore, it cannot be ruled out that the effects were attributable to other specific characteristics of the samples. Furthermore, specific type and amount of prior knowledge were not assessed and might have varied in the prior knowledge group. As such, we decided to manipulate participants’ expectations about the effects of Recall+EM in a second experiment. We first considered inducing positive expectations in one group and comparing effects of Recall+EM vs. Recall Only with a control group without prior knowledge or expectations. However, a stronger test would be to see if the positive effects of Recall+EM would survive a manipulation of participants’ knowledge of the proposed working mechanism of EMDR. Therefore, one group was told that, as a result of EM, memories should become less vivid and emotional (correct information) and the other that, as a result of EM, memories should become *more* vivid and emotional (incorrect information). As in Experiment 1, we measured memory vividness and emotionality before and after the Recall+EM and Recall Only interventions.

## 3. Experiment 2

### 3.1. Methods

#### 3.1.1. Participants

Forty-two participants were tested in Experiment 2. Inclusion was limited to individuals over 18 without current psychopathology, who reported to never have received EMDR therapy, and reported to have no specific knowledge of the memory degrading effects of EMDR and/or its proposed underlying working mechanism (similar to participants in the no knowledge group of Experiment 1). Two participants were excluded from the analyses because the pretest emotionality scores of their selected memories deviated ≥ 2.5 *SD* from the mean (Ratcliff, 1993) and low arousing, emotionally neutral memories have been found to be insensitive to the Recall+EM intervention (Littel, Remijn, Engelhard, & van den Hout, 2017; van den Hout, Eidhof, Verboom, Littel, & Engelhard, 2014). The final sample comprised 40 participants (*M*_age_ = 21.85, *SD*_age_ = 3.51, 13 males, 27 females). They were randomly assigned to a ‘correct information group’ (*n* = 20) or an ‘incorrect information group’ (*n* = 20). The two groups did not differ with regard to any of the measured demographic variables (see Table 4). All participants provided written informed consent and received course credit or financial compensation for participation.

**Table 4.** Demographics per information group.

	Correct information	Incorrect information
*n*	20	20
Mean age (*SD*)	21.00 (2.58)	22.70 (4.14)
Gender (male, female)	6, 14	7, 13
Ethnicity (Dutch, other)	16, 4	19, 1
Education (higher, lower)	19, 1	20, 1
Psychology student (yes, no)	8, 12	7, 13

#### 3.1.2. Materials and procedure

Prior to the experiment, participants were given information about EMDR. All participants were correctly informed that EMDR is used to treat PTSD, that it is highly effective, and that it is often called ‘a wonder therapy’, but that researchers are only just beginning to investigate how it works. Participants in the correct information condition were then told that this research repeatedly shows that, due to EMDR, traumatic memories become less vivid, less clear, and less accessible, and that, consequently, the memories become less unpleasant. Participants in the incorrect information condition were falsely explained that research repeatedly shows that, as a consequence of EMDR, traumatic memories become more vivid, clearer, and better accessible, and that, because of this, the memories temporarily become more unpleasant. However, because memories temporarily become better available, people can process them better in the long run.

After having received correct or incorrect information about the working mechanism of EMDR, participants were instructed to select two aversive, autobiographical memories. Then, participants proceeded to the dual taxation task, during which they recalled their memories while making EM (Recall+EM) or keeping eyes stationary (Recall Only). Before and after the interventions, memories were scored on vividness and emotionality (cf. procedure Experiment 1). Finally, as a retrospective manipulation check, the participants indicated how credible they found the provided information about the working mechanism of EMDR, both *before* and *after* they had received the EM intervention. They used 10 cm Visual Analog Scales (VASs) ranging from 0 (not very credible) to 100 (very credible).

#### 3.1.3. Data analysis

Data were again analysed with the BIEMS software package, using a Bayesian model selection criterion (Mulder et al., 2009, 2010, 2012).

Three hypotheses were compared in the analysis of self-reported credibility of the provided correct and incorrect information before and after the EM intervention: (1) both types of information are equally credible at first, but after the EM intervention the correct information becomes more credible; (2) the correct information is more credible than the incorrect information at first, which is still the case after the EM intervention; and (3) both types of information are equally credible at first, and remain so after the EM intervention. See Table 5 for the constraints of the three hypotheses.

**Table 5.** Hypothesis constraints for the manipulation check concerning the credibility of the correct and incorrect information. Pre = pretest, Post = posttest.

Hypothesis 1	Pre_Credibility _correct_ = Pre_Credibility _incorrect_ (Post_Credibility _correct_ – Pre_Credibility _correct_) > (Post_Credibility _incorrect_ – Pre_Credibility _incorrect_) Post_Credibility _correct_ > Post_Credibility _incorrect_
Hypothesis 2	Pre_Credibility _correct_ > Pre_Credibility _incorrect_ (Post_Credibility _correct_ – Pre_Credibility _correct_) > (Post_Credibility _incorrect_ – Pre_Credibility _incorrect_) Post_Credibility _correct_ > Post_Credibility _incorrect_
Hypothesis 3	Pre_Credibility _correct_ = Pre_Credibility _incorrect_ (Post_Credibility _correct_ – Pre_Credibility _correct_) = (Post_Credibility _incorrect_ – Pre_Credibility _incorrect_) Post_Credibility _correct_ = Post_Credibility _incorrect_

The main analysis of memory vividness and emotionality concerned two groups (correct and incorrect information), and two intervention conditions (Recall+EM and Recall Only). Pre-post change scores were calculated for vividness and emotionality ratings in Recall+EM and Recall Only conditions, with higher scores indicating larger decreases over time. Here, two hypotheses were compared: (1) providing information on the working mechanism of EMDR affects changes in memory vividness and emotionality after EM vs. Recall Only, with larger decreases after correct information than incorrect information; and (2) providing such information does not affect the effects of EM vs. Recall Only on memory vividness and emotionality. See Table 6 for the constraints of both hypotheses.

**Table 6.** Hypothesis constraints for vividness and emotionality (pre minus post) difference scores for the hypotheses concerning experimentally manipulated correct and incorrect information. EM = Eye Movements. There are no constrained hypotheses for incorrect information, because there was no a prior expectation of the effect.

Hypothesis 1	EM _correct_ > Recall Only _correct_
	(EM _correct_ – Recall Only _correct_) > (EM _incorrect_ – Recall Only _incorrect_)
Hypothesis 2	EM _correct_ > Recall Only _correct_
	(EM _correct_ – Recall Only _correct_) = (EM _incorrect_ – Recall Only _incorrect_)

### 3.2. Results

#### 3.2.1. Manipulation check for credibility

**Figure 2.**
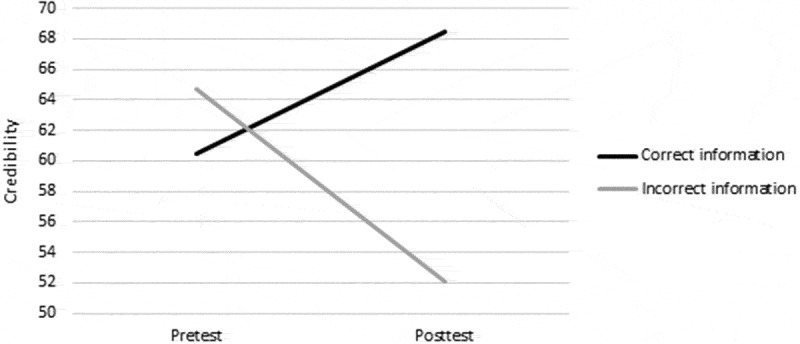
Mean credibility scores of the provided correct and incorrect information before (pretest) and after the eye movement intervention (posttest).

Results of self-reported credibility of the provided information on the working mechanism of EMDR correspond best with model 1. As can be seen in Figure 2, participants assessed the correct and incorrect information as approximately equally credible. Mean scores are 60.50 (*SD* = 19.78) and 64.71 (*SD* = 20.58) for correct and incorrect information respectively on VASs ranging from 0–100. However, a discrepancy arises after the intervention. After having experienced Recall+EM, the correct information becomes more credible (*M* = 68.47, *SD* = 19.34), whereas the incorrect information becomes less credible (*M* = 52.09, *SD* = 23.33). In accordance with these observed data patterns, Bayesian analysis shows a BF of 3.38 for model 1, which outperforms models 2 and 3 that have BFs of 1.87 and .23 respectively.

#### 3.2.2. Vividness and emotionality

**Figure 3.**
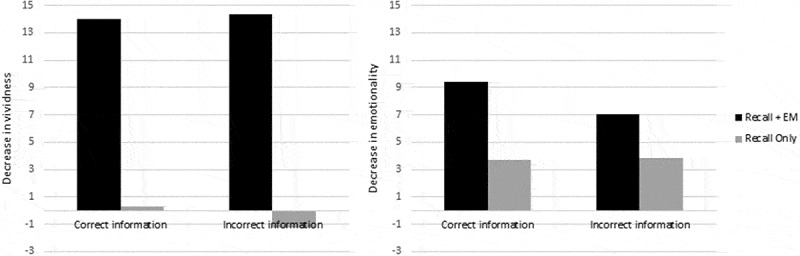
Mean decreases in memory vividness (left) and emotionality (right) after Recall+EM and Recall Only for the correct information and the incorrect information group.

As can be seen in Figure 3, there is a drop in memory vividness after Recall+EM relative to Recall Only. There appear to be no or only small differences between correct and incorrect information groups. The difference score (i.e., the decrease after Recall+EM minus decrease after Recall Only) for the correct group (*M* *_dif_* = 13.68) is only slightly smaller than for the incorrect information group (*M* *_dif_* = 15.64).

In addition, a drop in memory emotionality can be observed after Recall+EM relative to Recall Only. Again, there appear to be no or only small differences between correct (*M* *_dif_* = 5.73) and incorrect information groups (*M* *_dif_* = 3.19). In line with the observed data patterns, Bayesian analyses showed BFs of only 1.17 and 1.77 for vividness and emotionality for hypothesis 1, but BFs of 4.07 and 3.95 for hypothesis 2. Overall, hypothesis 2 outperforms hypothesis 1. Given the data, hypothesis 2 is 3.5 times more likely than hypothesis 1 for memory vividness and 2.2 times more likely for memory emotionality. Raw mean (*SD*) vividness and emotionality decreases can be found in the Appendix.

## 4. Discussion

Using a randomized design, it was demonstrated that providing information about the mechanism of EMDR to individuals who have no prior knowledge of EMDR did not increase the degrading effects of EM on the vividness and emotionality of their memories. Furthermore, the effects of Recall+EM vs. Recall Only survived the induction of negative expectations.

Importantly, these findings could not be attributed to the credibility of the information. The correct and incorrect descriptions of EMDR mechanism were found highly (and equally) credible at the start of the experiment. Interestingly, the correct information became *more* credible after the EM intervention, whereas the incorrect information became *less* credible. Participants might therefore be aware that their memories become less vivid and emotional due to EM. It must be noted that the credibility of the provided information was assessed retrospectively, and therefore might have been biased by the effects of the intervention. Assessing credibility directly after giving the information was not possible, as it could have caused participants to question the authenticity of the information. Nevertheless, despite retrospective assessment, credibility still changed from pretest to posttest.

Experiment 2 replicated the observation that Recall+EM is effective in reducing memory vividness and emotionality. It also showed that this EM effect is minimally affected by a priori raised expectations. Even when participants were told and expected that memories become *more* vivid and *more* emotional after making EM, they still reported strong reductions in vividness and emotionality. The participants started to question the provided information, whereas they believed it at first. These results contrast the view held by several previous authors that beneficial effects of EMDR treatment are incidental and can be explained by credibility, expectancy, or experimental demand (Devilly, 2005; Herbert et al., 2000; Lohr et al., 1992, 1999).

## 5. General discussion

We investigated whether expectations about the underlying mechanism of EMDR would alter the commonly observed, memory degrading effects of Recall+EM using two experiments: in the first we examined role of pre-existing knowledge, in the second we experimentally manipulated prior knowledge. Overall, data provided more evidence for the hypothesis that knowledge of the working mechanism of EMDR does not influence its effects. As observed in Experiment 2, providing information prior to an EMDR lab experiment does not affect the memory degrading effects of Recall+EM. However, as seen in Experiment 1, previously obtained knowledge of the mechanism of EMDR could boost the effects of Recall+EM. Because we did not find compelling evidence for one hypothesis over the other, the influence of prior knowledge seems to be relatively modest.

These results are highly relevant to the clinical practice. For many EMDR therapists it is common practice to explain to their patients how EMDR works and what to expect, thereby raising expectations (Shapiro & Forrest, 2016). Other therapists do the opposite and make very clear to the patients *not* to expect anything, as not experiencing an expected treatment outcome might be counter-therapeutic and lead to drop-out from therapy. The current results indicate that both strategies presumably have little impact, if any, on the beneficial effects of EMDR on emotional memories.

Although the BFs reported here indicate to what extent the current data supports one hypothesis over the other, it must be noted that the BFs (range 3.40–4.99) are not substantial or decisive, but only indicate ‘moderate support’ (Kass & Raftery, 1995). Therefore, replication or Bayesian updating with new data is recommended (Konijn, van de Schoot, Winter, & Ferguson, 2015). Furthermore, results should be replicated or updated in a treatment setting. It is possible that positive demand characteristics might affect memory attenuation differently compared to a laboratory setting.

To summarize, results of the present study indicate that memory Recall+EM decreases memory vividness and emotionality to a greater extent than Recall Only, and that it is quite robust against the effects of prior expectations. As Recall+EM is the core component of EMDR, it can be speculated that credibility and expectancy effects contribute little to the effectiveness of EMDR treatment.

## Highlights

1. Pre-existing knowledge of the working mechanism of EMDR has a relatively small impact on the memory degrading effects of memory recall + eye movements.
2. Providing participants with knowledge of the working mechanism of EMDR does not affect the memory degrading effects of memory recall + eye movements.
3. Recall + eye movements, the core intervention in EMDR, appears to be quite robust against prior expectations.

## Appendix

**Table:** Mean (SD) decreases (pretest minus posttest) in vividness and emotionality after Recall+EM and Recall Only in the two groups of Experiment 1 (prior and no knowledge) and Experiment 2 (correct and incorrect information). EM = eye movements.

		Prior knowledge	No knowledge	Correct information	Incorrect information
Vividness	Recall+EM	22.01 (21.75)	12.79 (30.60)	13.98 (23.17)	14.38 (23.16)
	Recall Only	3.27 (14.97)	.61 (17.83)	.30 (16.59)	–1.26 (15.09)
Emotionality	Recall+EM	22.60 (21.03)	8.80 (19.10)	9.43 (14.61)	7.01 (12.94)
	Recall Only	11.59 (16.10)	–2.27 (14.44)	3.70 (14.48)	3.82 (11.85)
